# Selective adsorption and determination of hexavalent chromium ions using graphene oxide modified with amino silanes

**DOI:** 10.1007/s00604-017-2640-2

**Published:** 2018-01-16

**Authors:** Paulina Janik, Beata Zawisza, Ewa Talik, Rafal Sitko

**Affiliations:** 1 Institute of Chemistry, University of Silesia, ul. Szkolna 9, 40-006 Katowice, Poland; 2 Institute of Physics, University of Silesia, ul. Uniwersytecka 4, 40-007 Katowice, Poland

**Keywords:** Speciation, Nanomaterial, Silanization, Solid-phase extraction, SPE, Dispersive micro-solid phase extraction, DMSPE, Chromate, Dichromate, Removal

## Abstract

Novel adsorbents are described for the preconcentration of chromium(VI). Graphene oxide (GO) was modified with various amino silanes containing one, two, or three nitrogen atoms in the molecule. These include 3-aminopropyltriethoxysilane (APTES), N-(3-trimethoxysilylpropyl)ethylenediamine (TMSPEDA), and N^1^-(3-trimethoxysilylpropyl)diethylenetriamine (TMSPDETA). The resulting GO derivatives were characterized by scanning electron microscopy, X-ray photoelectron spectroscopy, and energy-dispersive X-ray fluorescence spectrometry (EDXRF). Adsorption studies show that these GO based sorbents are highly selective for Cr(VI) in the presence of Cr(III) at pH 3.5. Although the amino silanes applied in modification of GO contain different numbers of nitrogen atoms, the maximum adsorption capacities of GO derivatives are very similar (13.3–15.1 mg·g^−1^). Such results are in accordance with spectroscopy studies which show that the amount of amino silanes attached to GO decreases in the order of APTES > TMSPEDA > TMSPDETA. The APTES-modified GO was applied to selective and sensitive extraction of Cr(VI) ions prior to quantitation by low-power EDXRF using the Cr K_α_ line. The Cr(VI) ions need not be eluted from the solid adsorbent. The method has a 0.17 ng·mL^−1^ detection limit, and the recovery is 99.7 ± 2.2% at a spiking level of 10 ng·mL^−1^. The method was successfully applied to the determination of Cr(VI) in water samples.

**Electronic supplementary material:**

The online version of this article (10.1007/s00604-017-2640-2) contains supplementary material, which is available to authorized users.

**Graphical abstract:**
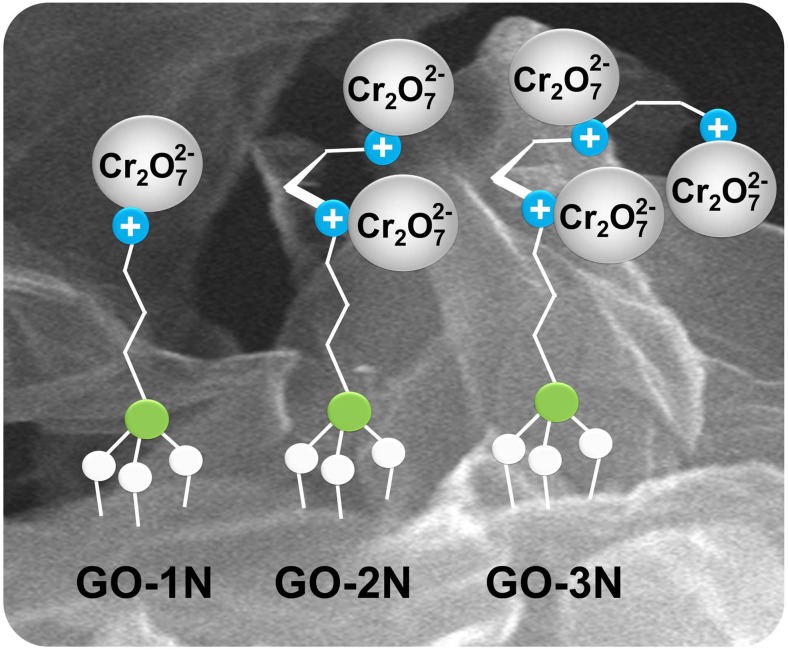
Graphene oxide adsorbents modified with various amino silanes are described for the preconcentration and speciation of trace and ultratrace levels of chromium ions.

## Introduction

Chromium is one of the environmental contaminant generated from industrial processes, such as leather tanning, dying, metallurgy, mining, and electroplating. Cr(III) and Cr(VI) are two main forms of chromium which exist in the water system. Both have a significant influence on human health: Cr(III) is an essential ion, whereas Cr(VI) compounds are highly toxic, mutagenic and carcinogenic. Taking this into consideration, it is very important to control hexavalent chromium (chromate) concentration in water. For this reason, the World Health Organization (WHO) has established an upper concentration limit for chromium in drinking water, which equals 50 ng⋅mL^−1^ [1]. Therefore, the development of new and sensitive analytical methods for chromium speciation in water samples is well-founded. Techniques which are used most commonly for the determination of Cr(VI) species are flame atomic absorption spectrometry (FAAS) [2, 3], electrothermal atomic absorption spectrometry (ETAAS) [4, 5], inductively coupled plasma optical emission spectrometry (ICP-OES) [6, 7], inductively coupled plasma mass spectrometry (ICP-MS) [8, 9], and X-ray fluorescence spectrometry (XRF) [10, 11]. Although, some of these techniques, e.g. ET-AAS or ICP-MS, offer excellent sensitivity and very low detection limits, the chromium speciation requires a preliminary separation of Cr(III) and Cr(VI). Apart from the hyphenated chromatographic techniques [12], the classical solid-phase extraction (SPE) and its miniaturized versions, such as solid-phase microextraction (SPME) or dispersive micro-solid phase extraction (DMSPE), are the most commonly employed techniques for the preconcentration/separation of chromium species. In these techniques, suitable selection of adsorbent is crucial to ensure quantitative retention of the trace elements and, in some cases, selective adsorption. The carbon-based nanomaterials, i.e. carbon nanotubes (CNTs), graphene and graphene oxide (GO), have become more important in SPME or DMSPE due to their excellent properties [13, 14]. However, they are not selective toward metal ions. The improvement of their selectivity can be realized by application of surfactant [15–17], suitable chelating agent [18, 19], or through chemical functionalization, e.g. via silanization [20–22] or creation of ester or amide bonds [23, 24]. In case of Cr(VI), the amino-functionalized GO seems to be the appropriate adsorbent because the protonated amino groups provide a positive surface charge that is suitable for the adsorption of Cr(VI) anions [25]. In comparison with non-modified GO, the point of zero charge (pH_PZC_) of amino-derivatives is shifted to the neutral position (pH 6–8). Therefore, the high adsorption of Cr(VI) is observed at pH lower than 6–8. The non-modified GO (pH_PZC_ value of ca. 4) is much more suitable for adsorption of Cr(III) rather than Cr(VI). The adsorption of Cr(III) is observed at pH > 4 [10], whereas, the adsorption of Cr(VI) requires strong acidic solution, and it is not usually quantitative.

In the present work, GO was modified with three different amino silanes containing one, two and three nitrogen atoms in the molecule (GO-1 N, GO-2 N and GO-3 N) for selective adsorption of Cr(VI) ions. The amino-silanized derivatives of GO were characterized by microscopy and spectroscopy techniques. The adsorption of Cr(VI) on modified GO was investigated by batch adsorption experiment including influence of pH, kinetics, and Langmuir and Freundlich isotherm models. GO-1 N was used as an effective adsorbent in DMSPE in order to determine trace amounts of Cr(VI) ions by the energy-dispersive X-ray fluorescence spectrometry (EDXRF). Chromium was quantified directly on membrane, therefore, the Cr(VI) ions did not have to be eluted from the solid adsorbent. The method was applied for the determination of Cr(VI) in various types of environmental water samples.

## Experimental

### Reagents and solutions

Stock solutions (1 mg·mL^−1^ of Cr(III) and Cr(VI)), (3-aminopropyl)triethoxysilane (APTES, 99%), N-[3-(trimethoxysilyl)propyl]ethylenediamine (TMSPEDA, 97%) and N^1^-(3-trimethoxysilylpropyl)diethylenetriamine (TMSPDETA) were purchased from Sigma-Aldrich (Steinheim, Germany, www.sigmaaldrich.com); nitric acid (65%, p.a.), sulfuric acid (98%, p.a.), ethanol (p.a.), ammonium hydroxide solution (25%, p.a.), potassium permanganate (p.a.), sodium nitrite (p.a.) were from POCh (Gliwice, Poland, www.poch.com.pl). Standard solutions were diluted with high purity water obtained from Milli-Q system (Millipore, Molsheim, France, www.merckmillipore.com).

### Instruments

The microstructural observations of the GO derivatives were conducted using JEOL-7600F (www.jeol.co.jp/en) scanning electron microscope (SEM) equipped with the Oxford X-ray energy-dispersive spectrometer (EDS). The chemical composition was confirmed by X-ray photoelectron spectroscopy (XPS). Photoelectron spectra were collected using a PHI 5700/660 Physical Electronic spectrometer (www.phi.com) with monochromated Al Kα radiation. The spectra were analyzed with a hemispherical mirror assuring an energy resolution of about 0.3 eV. Three hours after placing the samples in situ at 10^−10^ hPa vacuum, their surface was clean enough for measurements. The background was subtracted using the Tougaard’s approximation. The EDXRF measurements (chemical composition of GO derivatives and determination of Cr adsorbed on GO derivatives) were performed using an Epsilon 3 spectrometer (Panalytical, Almelo, The Netherlands, www.panalytical.com), equipped with Rh target X-ray tube of a 50 μm Be window and maximum power of 9 W. The X-ray spectra were collected using a thermoelectrically cooled silicon drift detector (SDD) with 8 μm Be window and 135 eV resolution at 5.9 keV. The system is also equipped with 10-position removable sample changer, spinner and five primary filters that can be selected to improve measuring conditions for determined element. Evaluation of spectra was performed using non-linear least squares fitting, based on the AXIL algorithm (Epsilon 3 software). The concentration of Cr in solutions (batch adsorption experiments: pH effect, isotherms, kinetics, sample volume) was determined by ICP-OES using Spectroblue spectrometer (Spectro Analytical Instruments GmbH, Germany, www.spectro.com) and following parameters: polychromator with focal length 750 mm, holographic master grating, wavelength range 165–770 nm; detector: 15 linear CCD arrays, 3648 pixels per array; generator: frequency 27.12 MHz, power 0.7–1.7 kW, air cooled; nebulizer Cross Flow type; exhaust system requirements: torch box: 200–300 m^3^⋅h^−1^, generator: 250–300 m^3^⋅h^−1^. The wavelength for Cr was 267.716 nm.

### Synthesis of GO derivatives

GO was synthesized by the Hummers method. The GO was modified with the molecules containing different number of nitrogen atoms, i.e. APTES, TMSPEDA and TMSPDETA: (C_2_H_5_O)_3_Si(CH_2_)_3_NH_2_, (CH_3_O)_3_Si(CH_2_)_3_NH(CH_2_)_2_NH_2_ and (CH_3_O)_3_Si(CH_2_)_3_NH(CH_2_)_2_NH(CH_2_)_2_NH_2_. GO derivatives were denoted as GO-1 N, GO-2 N and GO-3 N, respectively. The synthesis of GO, GO-1 N, GO-2 N and GO-3 N is described in detail in Electronic Supplementary Materials.

### Adsorption procedure

The batch adsorption experiments were carried out with 5 mg of GO derivatives (GO-1 N, GO-2 N or GO-3 N) and 25 mL of Cr(VI) aqueous solutions with the desired concentration and pH. The pH values of the suspensions were adjusted with nitric acid or ammonia solutions. Then, the suspensions were stirred for 3.0 h to achieve adsorption equilibrium (in the case of adsorption isotherms) or stirred within 30–300 min (kinetic study). The suspensions were filtered through a 0.45 μm membrane filters. The amount of Cr(VI) ions adsorbed on GO derivative *q*_*e*_ or *q*_*t*_ (mg⋅g^−1^) was calculated from the difference between the initial concentration *C*_*0*_ (mg⋅L^−1^) and equilibrium concentration *C*_*e*_ (mg⋅L^−1^) determined by ICP-OES after filtration: *q*_*e*_ = (*C*_*0*_ − *C*_*e*_)*V*/*m*_*adsorbent*_, where *V* is the volume of the suspension, and *m*_*adsorbent*_ is the mass of GO derivative.

### Preconcentration procedure

The pH value of samples (50 mL) was adjusted to 3.5 using 0.1 mol⋅L^−1^ HNO_3_ and/or 0.1 mol⋅L^−1^ NH_3_. Then, 1 mL of GO-1 N suspension (5 mg⋅mL^−1^ GO-1 N) was injected to the analyzed solution and stirred for 180 min. In the next step, the sample was passed through the membrane filter with the use of filtration assembly of 25 mm in diameter (deposit of GO-1 N on membrane filter of 16 mm in diameter). Subsequently, the membrane filter with GO-1 N and Cr(VI) adsorbed on its surface was dried under an IR heater and measured by EDXRF.

### Real samples preparation

All environmental water samples were filtered through a Millipore cellulose acetate membrane filter (0.45 μm), acidified with concentrated nitric acid (pH 2) and stored in polyethylene bottles at 4 °C.

## Results and discussion

### Choice of materials

GO was selected for modification from among many adsorbents for two main reasons: (i) it possess large quantities of functional groups (much higher than oxidized CNTs [14]) that can be modified with amino silanes, (ii) it has serious advantages in the view of EDXRF measurements. The second reason was particularly important because adsorbent collected onto the membrane was directly measured without eluting step. In contrast to nano-oxides (including magnetic nanoparticles), GO contains only carbon, oxygen and hydrogen atoms. Therefore, characteristic radiation of adsorbent is not visible in EDXRF spectrum. Due to the low atomic number of carbon, oxygen, hydrogen, the attenuation of X-ray fluorescent radiation of chromium is very low. Moreover, such features of GO nanosheets as flexibility and softness allow obtaining smooth and durable thin sample after filtration process. These reasons make GO ideal adsorbent for direct EDXRF analysis. GO was modified with amino silanes containing amino groups that can be protonated in acidic solution and are responsible for interaction with anionic species of Cr(VI). The chemical modification significantly improves adsorptive properties of GO toward Cr(VI) but does not worsen its properties in the view of EDXRF measurement (characteristic radiation of nitrogen is not visible in EDXRF spectrum; Si Kα line can be observed in the low-energy EDXRF spectrum).

### Characterization of GO-1 N, GO-2 N and GO-3 N

**Fig. 1:**
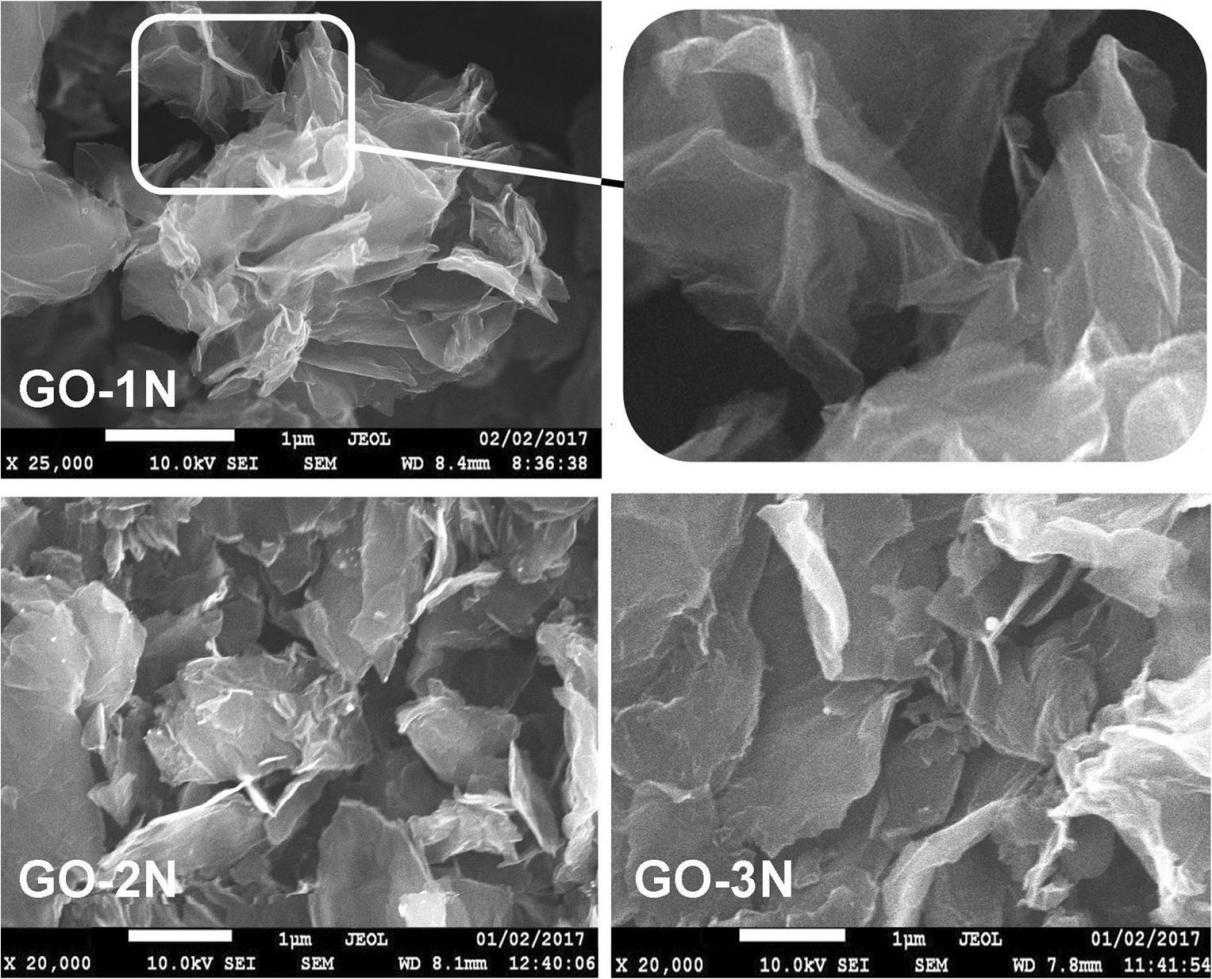
SEM images of GO-1 N, GO-2 N and GO-3 N

Amino-silanized GO derivatives were characterized by SEM/EDS, EDXRF and XPS. The SEM images presented in Fig. 1 show the highly wrinkled structure of GO derivatives. This structure in concert with the softness and flexibility of nanosheets allow achieving excellent contact with the analyzed solution. SEM/EDS analysis reveals that GO-1 N, GO-2 N and GO-3 N contain 4.3 ± 0.22, 4.3 ± 0.42 and 4.9 ± 0.14% N, respectively. Very similar concentration of N in GO derivatives suggests that TMSPDETA was attached to GO nanosheets in lower degree than TMSPEDA, and TMSPEDA lower than APTES, although GO-1 N, GO-2 N and GO-3 N were synthesized using the same amount of amino silanes. EDXRF analysis (Fig. S1a) confirms that the Si concentration decreases in the order of GO-1 N, GO-2 N and GO-3 N. The synthesized GO-1 N was also characterized by XPS. The results are discussed in Electronic Supplementary Materials (Fig. S1b).

### Adsorption of chromium ions on GO-1 N, GO-2 N and GO-3 N

**Fig. 2:**
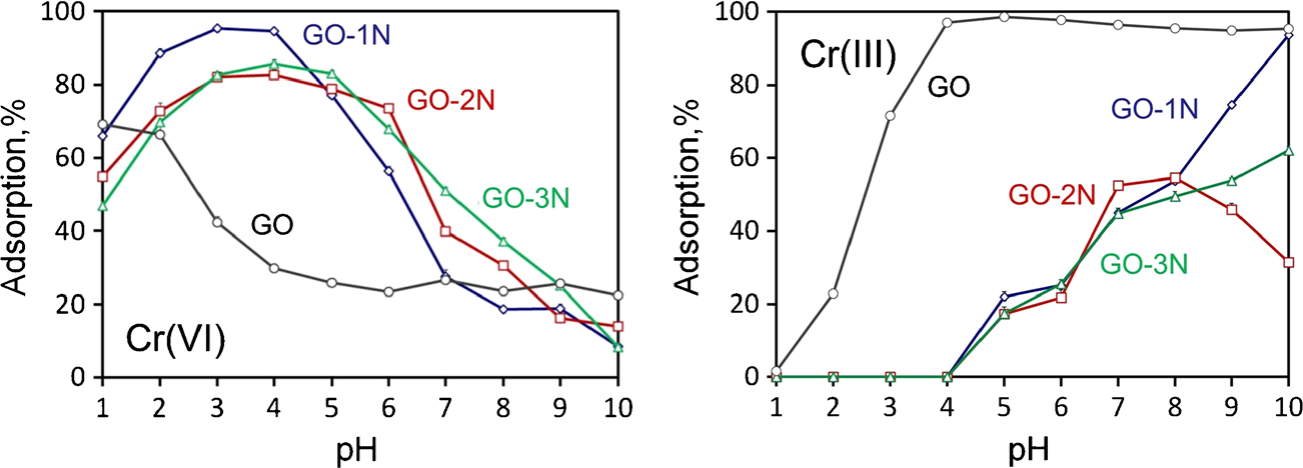
Influence of pH on adsorption of Cr(VI) and Cr(III) on GO, GO-1 N, GO-2 N and GO-3 N; adsorption conditions: pH = 3.5, *T* = 25 °C, C_0_ = 0.25 mg⋅L^−1^, V = 25 mL, m_adsorbent_ = 5 mg, *t* = 180 min

The acidity of sample solution is one of the most important parameters in the adsorption of metal ions. The acidity influences not only the adsorbent surface charge but also the metal species present in solution. The adsorption of Cr(VI) ions on GO modified with three different silanes, such as: APTES, TMSPEDA and TMSPDETA was investigated at pH ranging from 1 to 10. The experimental results for GO-1 N, shown in Fig. 2, indicate that adsorption of Cr(VI) increases at pH ranging from 1 to 3, remains constant at pH from 3 to 4, and decreases at pH 4–10. The maximum adsorption for GO-2 N and GO-3 N is shifted to a little higher pH value. At low pH, Cr(VI) assumes the anionic form, such as HCrO_4_^−^, Cr_2_O_7_^2−^ and CrO_4_^2−^. Simultaneously, the large quantities of protons in acid solution can protonate amino groups onto the surface of modified GO, that are responsible for electrostatic interaction with anionic species of Cr(VI). Additionally, hydrogen bond interaction between HCrO_4_^−^ and functional groups (Si-OH and -NH_3_^+^) of amino-modified GO can be formed [26, 27]. In basic solution, the negative charge is generated on the surface of adsorbent and electrostatic attraction is not possible. Taking into account the high adsorption of Cr(VI), pH 3.5 was chosen as the optimum pH for further studies (kinetics, adsorption isotherms, and determination of chromium by EDXRF). Adsorption of other metal ions at pH 3.5 is very low or even close to zero (Fig. S2). Fig. 2 also shows the effect of pH on the adsorption of Cr(VI) ions on the non-modified GO. As can be noticed, the maximum adsorption is shifted to acidic solution because GO is stronger acid than its amino-derivatives (pKa of -COOH in the range of 4–5, and pKa of -NH_3_^+^ in the range of 7–8 [25]). The efficiency of adsorption of Cr(VI) on GO decreases with the increase of sample pH. The highest value was achieved at pH 1, however, the adsorption is not quantitative. In strong acidic solution, Cr(VI) can be partially reduced to Cr(III): HCrO_4_^−^ + 7H^+^ + 3e^−^ → Cr^3+^ + 4H_2_O [28], which is not adsorbed on positively charged surface of GO, GO-1 N, GO-2 N and GO-3 N. The influence of pH on the adsorption of Cr(III) was also studied. As can be seen in Fig. 2, the adsorption is close to zero at pH 1–6. Such values can be explained by electrostatic repulsion between ionic species of Cr(III) such as: Cr(H_2_O)_6_^3+^, Cr(OH)(H_2_O)_5_^2+^, Cr(OH)_2_(H_2_O)_4_^+^ and the -NH_3_^+^ groups that are predominant at acidic solutions. At pH higher than 7 the adsorption % increases, because Cr(III) ions can be chelated through functional groups, as well as, can begin to precipitate as Cr(OH)_3_. In case of GO the adsorption increases quickly at pH 1–4 and remains constant in broad range of pH from 4 to 10. This is a result of strongly electrostatic interaction between cationic form of Cr(III) and negative surface charge of GO. Besides, Cr(III) ions can be chelated through functional groups on the GO surface.

The adsorption significantly depends on the adsorption time and sample volume. The effect of the sorption time and sample volume on the adsorption of Cr(VI) was investigated for 20–100 mL sample volume during 30–300 min. Fig. S3 shows that adsorption of Cr(VI) on the GO-1 N, GO-2 N and GO-3 N surfaces increases quickly at the beginning of the experiment and then reaches the equilibrium state. The adsorption decreases with increasing sample volume. As can be noticed, quantitative adsorption was achieved in the range of stirring time from 180 to 300 min for examined volumes of sample. From a practical point of view, the analysis of real water samples was performed using the sample volume of 50 mL and sorption time of 180 min. It is also noteworthy here that a dozen samples can be simultaneously prepared using one multiposition magnetic stirrer.

The kinetic data (the pseudo-second order rate adsorption kinetic model, Table S1, Fig. S3) and adsorption isotherms (Langmuir and Freundlich isotherm models, Table S2, Fig. S4) suggest that adsorption of Cr(VI) on GO-1 N, GO-2 N and GO-3 N nanosheets is monolayer coverage and it is controlled by electrostatic interaction between anionic species of Cr(VI) and protonated amino groups onto the surface of GO derivatives. The adsorption capacity is proportional to the number of active sites occupied on the surface of modified adsorbents. The maximum adsorption capacities *q*_*max*_ of Cr(VI) on GO-1 N, GO-2 N and GO-3 N are very similar and equal 13.3, 15.1, 14.3 mg⋅g^−1^, respectively. Such results are in accordance with SEM/EDS analysis that reveals very similar contents of N in GO-1 N, GO-2 N and GO-3 N, i.e. the same number of active sites on the surface of GO derivatives. GO-1 N was selected for determination of Cr(VI) because of the best recovery at pH 3.5 (Fig. 2).

### Application of GO-1 N in determination of trace Cr(VI) ions

The synthesized adsorbent was applied in determination of trace hexavalent chromium ions. The procedure is based on DMSPE and consists of following stages: (i) dispersing GO-1 N in aqueous sample adjusted to pH 3.5 and stirring, (ii), collecting GO-1 N with adsorbed Cr(VI) ions on membrane filter via vacuum filtration, (iii) measurement by EDXRF. The flexibility and softness of GO-1 N nanosheets allow preparing smooth and thin samples deposited onto a membrane, which is a crucial issue in XRF spectrometry. In order to study the analytical figures of merit of DMSPE/EDXRF procedure, the calibration curve was obtained by the preparation of a series of Cr(VI) standard solution. Results presented in Table 1 show the linear relationship between analytical signal (corrected peak area) and chromium concentration in aqueous samples in the range of 2–1400 ng·mL^−1^ range, which is reflected in a correlation coefficient R^2^ 0.9996 (Fig. S5). The recovery value for Cr(VI) is ca. 100%. The relative standard deviation (RSD) characterizing precision of the method is 2.2%, which can be considered as very good for trace analysis. The limit of detection (LOD) and limit of quantification (LOQ) were calculated from the following equations: LOD = (3·*k*^−1^)·(*R*_*B*_·*t*^−1^)^1/2^, where *k* is the sensitivity of the method, *R*_*B*_ is the background count rate in counts·s^−1^ and *t* is the counting time, and LOQ = 3·LOD. It’s worth to emphasize that the LOD is ca. 300 times lower than the acceptable maximum contaminant levels for chromium that were established by WHO [1] and the Polish regulations for mineral and bottled water (50 ng⋅mL^−1^) [29]. The Table 2 shows the comparison of the analytical methods for determination of Cr(VI) using various adsorbents including amino-functionalized materials. The value of LOD depends on detection method used for quantification of chromium. The lowest LODs can be achieved using ICP-OES, and, most of all, ET-AAS. However, the LOD of EDXRF method (0.17 ng⋅mL^−1^) was obtained with the use of very low-power instrument (9 W X-ray tube) with no gas consumption. Moreover, the DMSPE/EDXRF method requires very small amount of adsorbent, i.e. 100 μg per 1 mL of the liquid sample. Therefore, it can be considered as environmentally friendly and lies in the green analytical chemistry rules. The maximum adsorption capacity (*q*_*max*_ = 13.3 mg⋅g^−1^) is rather moderate. However, it is sufficient for determination of trace Cr(VI). The acidic solution (pH 3.5) is suitable for quantitative adsorption of Cr(VI) and simultaneously prevents the precipitation of some heavy metal ions present in real samples.

**Table 1:** Analytical figures of merit of the DMSPE/EDXRF procedure using GO-1 N as solid adsorbent; measurement conditions: Rh X-ray tube operated at 20 kV and 450 μA, 120 s counting time, air atmosphere, 200 μm Al primary beam filter

Parameter	Value
Recovery^a^, %	99.7 ± 2.2
RSD^a^, %	2.2
LOD, ng⋅mL^−1^	0.17
LOQ, ng⋅mL^−1^	0.51
Sensitivity, mL⋅ng^−1^⋅s^−1^	3.70 ± 0.032
Linear range, ng⋅mL^−1^	2 – 1400
Correlation coefficient R^2^	0.9996

^a^C = 10 ng⋅mL^−1^

**Table 2:** Comparison of different solid adsorbent in the determination of Cr(VI) by various spectroscopic techniques

Material	Type of modificator	Amount of adsorbent, mg	Sample volume, mL	pH	*q*_*max*_, mg⋅g^−1^	Detection technique	LOD, ng⋅mL^−1^	Matrix	Ref.
Fe_3_O_4_/SiO_2_	AAMDMS^*a*^	25	45	5.0	–	FAAS	1.1	Water samples	[2]
UVM-7^*b*^	APTES	120	100	2.0	172	FAAS	1.2	Water samples	[30]
Fe_3_O_4_@INPs^*c*^	APTES	500	500	3.0	2.5	FAAS	0.29	Water samples	[31]
Fe_3_O_4_@GO	TETA^*d*^	50	50	2.0	16	FAAS	1.4	Environmental water	[3]
SBA-15^*e*^	APTES	15	10	2.0	–	FAAS	0.2	Water samples	[32]
Fe_3_O_4_	PAEDTs^*f*^	14.8	400	2.0	–	ETAAS	0.001	Water and tea samples	[4]
Fe_3_O_4_@MnO_2_,Al_2_O_3_	AAPTMS^*g*^	250	5	6.0	30	ICP-OES	0.02	River water samples	[6]
CuNCs^*h*^	DAMP^*i*^	4uM	25	9.0	–	ICP-OES	0.016	Drinking, tap and groundwater	[7]
MCM-41^*j*^	APTMS^*k*^	25	100	9.0	111	ICP-OES	4	Water samples	[33]
TRG^*l*^	APTES	20	25	1.7	–	UV-Vis^*m*^	0.4	Tap, river, sewage and ground water	[34]
Fe_3_O_4_	PANI^*n*^	14.5	100	7.6	54	HPLC	0.1	Spiked water samples	[35]
CNTs	Aliquat 336^*o*^	5	20	2	–	TXRF^*p*^	3	Tap and mineral water	[36]
GO	APTES	5	50	3.5	13.3	EDXRF	0.17	Environmental water	This work

^*a*^2-aminoethyl-3-aminobutylmethyldimethoxysilane; ^*b*^bimodal mesoporous silica nanoparticles; ^*c*^magnetic Cr(VI)-imprinted nanoparticles; ^*d*^triethylenetetramine; ^*e*^mesoporous silica SBA-15; ^*f*^2-(propylaminoethyl)dithiocarbamate; ^*g*^3-(2-aminoethylamino)propyl]trimethoxysilane, ^*h*^ copper nanoclusters; ^*i*^4,6-diamino-2-mercaptopyrimidine; ^*j*^mesoporous silica MCM-41; ^*k*^3-aminopropyltrimethoxysilane; ^*l*^thermally reduced graphene; ^*m*^UV-Vis spectrophotometry; ^n^polyaniline; ^*o*^tricaprylmethylammonium chloride; ^*p*^total-reflection X-ray fluorescence spectrometry

The reliability of the procedure was verified by the analysis of five different types of natural water samples. The studies were carried out by the standard addition method on two levels of chromium concentration (10 and 20 ng⋅mL^−1^). The results presented in Table 3 show the recovery in the range of 89–100%. The advantage of the methodology is good resistance to sample matrix. Since GO-1 N is highly selective toward Cr(VI) at pH 3.5, the speciation analysis can also be performed (Table 4). The total concentration of Cr was calculated after oxidation of Cr(III) to Cr(VI) with KMnO_4_ according to procedure described in literature [37]. Then, the Cr(III) content was calculated as the difference between total chromium and Cr(VI) concentration. The results presented in Table 4 indicate that method can be successfully applied for speciation of chromium species in real water samples with good recovery and precision.

**Table 3:** Determination of Cr(VI) in spiked water samples; *n* = 3; uncertainties correspond to one standard deviation

Sample	Added, ng⋅mL^−1^	Found, ng⋅mL^−1^	Recovery, %
Tap water	0	< LOD	
	10	9.84 ± 0.06	98.4
	20	19.53 ± 0.01	97.6
Spring water	0	< LOD	
	10	9.73 ± 0.08	97.3
	20	20.0 ± 0.55	100.0
High mineral water	0	< LOD	
	10	9.3 ± 0.79	93.0
	20	17.9 ± 0.39	89.5
Lake water	0	< LOD	
	10	9.3 ± 0.83	93.0
	20	18.3 ± 0.51	91.5
Sea water	0	< LOD	
	10	9.0 ± 0.28	90.0
	20	18.4 ± 0.47	92.0

**Table 4:** Determination of Cr(III) and Cr(VI) in spiked water samples

Added, ng⋅mL^−1^		Found, ng⋅mL^−1^		Recovery, %	
Cr(III)	Cr(VI)	Cr(III)	Cr(VI)	Cr(III)	Cr(VI)
0	0	˂ LOD	˂ LOD		
0	10	˂ LOD	9.87 ± 0.016		98.7
10	0	9.25 ± 0.020	˂ LOD	92.5	
10	10	9.30 ± 0.044	9.48 ± 0.018	93.0	94.8

## Conclusions

Novel adsorbents were synthesized by modification of GO with three amino silanes, APTES, TMSPEDA, and TMSPDETA containing one, two and three nitrogen atoms in molecule, respectively. Although GO was modified with amino silane containing different number of nitrogen atoms, the maximum adsorption capacities of synthesized adsorbents were very similar. It suggests that the amount of amino silanes attached to GO nanosheets decreases in the order of APTES > TMSPEDA > TMSPDETA. Such results are in accordance with spectroscopy studies. The maximum adsorption capacity of 13.3–15.1 mg⋅g^−1^ is rather moderate. However, it is sufficient for determination of trace Cr(VI). The selectivity of GO derivatives toward Cr(VI) ions indicates their potential application in chromium determination and speciation. The quantitative adsorption requires time of 180 min. However, a dozen samples can be simultaneously prepared using one multiposition magnetic stirrer. The adsorbent is collected onto the membrane and measured directly by EDXRF. Therefore, the Cr(VI) ions do not have to be eluted from the solid adsorbent. Very low LOD of 0.17 ng⋅mL^−1^ was achieved with the use of very small amount of adsorbent (100 μg per 1 mL of aqueous sample) and very low-power instruments (9 W X-ray tube) with no gas consumption. Therefore, the method can be considered as environmentally friendly and lies in the green analytical chemistry rules: reduction in the use of reagents and energy, waste minimization.

## Electronic supplementary material

ESM 1 (PDF 808 kb)
