# Supplementary material for: Selective adsorption and determination of hexavalent chromium ions using graphene oxide modified with amino silanes
Source: Mikrochim Acta. 2018 Jan 16;185(2):117. doi: 10.1007/s00604-017-2640-2 (PMC5770511; doi:10.1007/s00604-017-2640-2)
Supplement: Supplementary file 1 — (PDF 808 kb) [file 604_2017_2640_MOESM1_ESM.pdf]

Electronic Supplementary Material

Selective adsorption and determination of hexavalent chromium ions using graphene oxide modified with amino silanes

Paulina Janik[a], Beata Zawisza[a], Ewa Talik[b], Rafal Sitko[a*]

[a] University of Silesia, Institute of Chemistry, ul. Szkolna 9, 40-006 Katowice, Poland
[b] University of Silesia, Institute of Physics, ul. Uniwersytecka 4, 40-007 Katowice, Poland

* Corresponding author: rafal.sitko@us.edu.pl

Synthesis of GO

GO was synthesized by the Hummers method [1]: 70 mL of concentrated H_2SO_4 were added to a mixture of graphite flakes (3.0 g) and $NaNO_3$ (1.5 g). The mixture was cooled to 0 °C and 9 g of $KMnO_4$ was added slowly in small portions to keep the reaction temperature below 20 °C. Then, the mixture was warmed to 35 °C and stirred for 12 h. The reaction mixture was cooled to room temperature and poured onto ice (400 mL) with 30% H_2O_2 (3 mL). Then the mixture was centrifuged at 4000 rpm (2612 rcf) within 5 min. The solid product was washed 20 times with water and 30 times with 5% HCl. Each time, the solid was redispersed by ultrasonication and collected by centrifugation. Then, the solid phase was rinsed with deionized water. The centrifugation and ultrasonication with a new portion of deionized water were repeated ca. 20 times until the solution was neutral. Then, the obtained GO was dried at 100°C. The derivatives (GO-1N, GO-2N and GO-3N) were synthesized as follows [2,3]: the suspension of 1 g of GO in 300 mL of anhydrous ethanol was sonicated for 1 h. Then, 10 mL of amino silanes (APTES, TMSPEDA or TMSPDETA) were added to the suspension. The mixture was heated to 70 °C in a water bath and refluxed for 4 h. The solid phase was collected by centrifugation and washed 8 times with ethanol and 8 times with water to remove the APTES, TMSPEDA and TMSPDETA residuals. Each time, the solid was redispersed by ultrasonication and collected by centrifugation. The obtained materials were dried at 100 °C.

Characterization of GO-1N, GO-2N and GO-3N

EDXRF analysis (Fig. S1a) shows that the Si concentration decreases in the order of GO-1N, GO-2N and GO-3N. The synthesized GO-1N was characterized by XPS. The high-resolution C1s spectra of non-modified GO and GO-1N are presented in Fig. S1b. The C1s spectrum of GO shows the peaks at 284.4 eV, 285.8, 286.9, 288.1 and 289.5 eV assigned to C-C/C-H, C-OH, C-O-C, C=O and O-C=O, respectively. The C1s spectrum of GO-1N was deconvoluted into six peaks at 283.1 eV (C-Si), 284.4 eV (C-C and C-H), 285.4 eV (C-OH, C-O-Si, C-N), 286.7 eV (C-O-C), 288.1 eV (C=O) and 289.5 eV (O-C=O) [3 - 7]. The presence of new peak at 283.1 eV (C-Si) and different peak intensities and binding energies for GO and GO-1N indicate the successful modification of GO nanosheets with APTES. The decrease in the epoxy and hydroxyl groups present on the surface of GO-1N, and simultaneously, new C-O-Si and C-N bonds contributing to the peak at 285.4 eV are observed [7]. The APTES is a bi-functional silane. Therefore, APTES can also be grafted to GO surface through a linkage between amine group in APTES and the carboxylic group on GO. However, the very small peak at 289.5 eV indicates that APTES is attached to GO through hydroxyl and epoxy groups on GO surface rather than through the amine linkage [6].

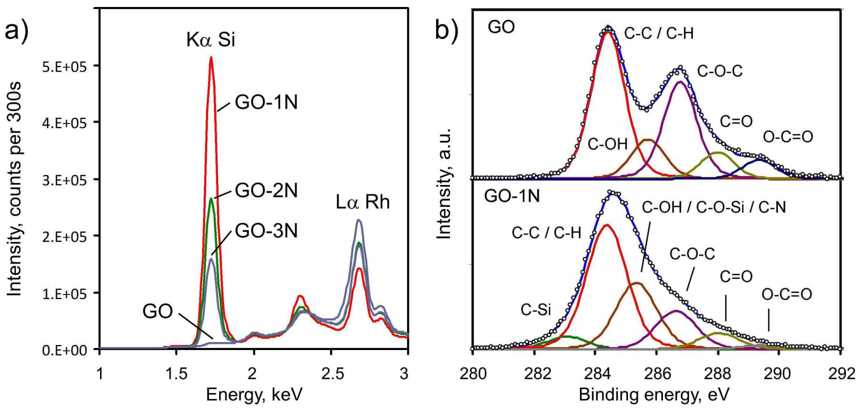

Fig. S1. EDXRF spectra of GO, GO-1N, GO-2N and GO-3N (a) and the C1s high-resolution XPS spectra of GO and GO-1N (b).

Influence of pH on adsorption of metal ions on GO-1N

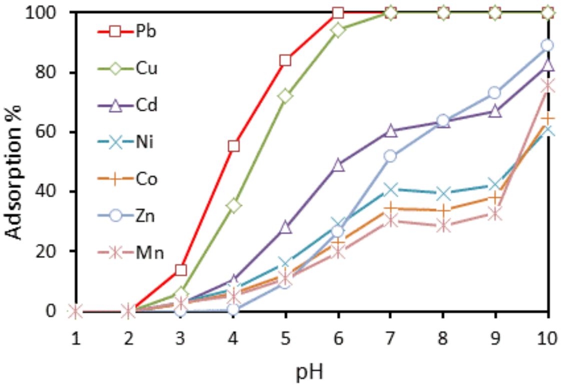

Fig. S2. Influence of pH on adsorption of metal ions on GO-1N; adsorption conditions: T = 25 ºC, C_0 = 0.25 mg·L^{-1}, V = 25 mL, $m_{adsorbent}$ = 5 mg, t = 180 min.

The effect of the sorption time and sample volume on the adsorption of Cr(VI)

The kinetic of an adsorption process was studied using the pseudo-second order rate adsorption kinetic model [8]:

$$\frac{1}{q_t} = \frac{1}{k_2 q_e^2} + \frac{t}{q_e}$$

where q_e and q_t (mg·g^{-1}) are the capacities of metal ions adsorbed at the equilibrium and time t (min), respectively, k_2 is the pseudo-second-order rate constant (g·mg^{-1}·min^{-1}). The calculated kinetic parameters for adsorption of Cr(VI) ions on GO-1N, GO-2N and GO-3N nanosheets are listed in Table S1. As can be seen, the experimental data for the adsorption of Cr(VI) ions are very well-fitted by the kinetics model (R = 0.992-1.000). Moreover, it can be perceived, that the pseudo-second-order rate constant decreases with the increase of sample volume. These results indicate that the adsorption of Cr(VI) on GO modified with three different amino silanes, is faster at a lower volume of samples (Fig. S3). It results from the higher adsorbent dosage in case o sample of low volume (0.25 and 0.05 g·L^{-1} for 20 and 100 mL sample, respectively). The experimental q_e (1.25 mg·g^{-1}) is close to q_e values calculated from the pseudo second-order kinetic model. The kinetic data suggest that adsorption of Cr(VI) on GO-1N, GO-2N and GO-3N nanosheets is monolayer coverage and it is controlled by electrostatic interaction between anionic species of Cr(VI) and protonated amino groups onto the surface of GO derivatives. The adsorption capacity is proportional to the number of active sites occupied on the surface of modified adsorbents.

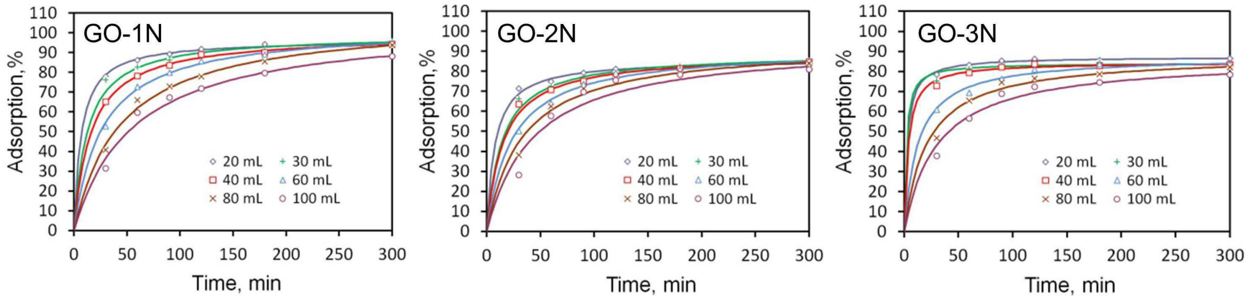

Fig. S3. Effect of sample volume and contact time; adsorption conditions: pH = 3.5, T = 25 ºC, 6.25 µg Cr(VI), V = 20 - 100 mL, $m_{adsorbent}$ = 5 mg, time = 30 - 300 min.

Table S1. The calculated kinetic parameters (pseudo-second order rate adsorption kinetic model) for adsorption of Cr(VI) ions on GO-1N, GO-2N and GO-3N nanosheets.

Adsorbent	Volume, mL	q_e, mg·g^{-1}	k_2, g·mg^{-1}·min^{-1}	r
GO-1N	20	1.206 ± 0.005	0.12 ± 0.02	1.0000
	30	1.24 ± 0.01	0.07 ± 0.01	0.9997
	40	1.239 ± 0.007	0.050 ± 0.003	0.9999
	60	1.288 ± 0.008	0.030 ± 0.001	0.9999
	80	1.34 ± 0.02	0.017 ± 0.001	0.9995
	100	1.31 ± 0.04	0.014 ± 0.001	0.9976
GO-2N	20	1.073 ± 0.004	0.13 ± 0.01	1.0000
	30	1.114 ± 0.009	0.065 ± 0.007	0.9998
	40	1.108 ± 0.008	0.060 ± 0.005	0.9999
	60	1.14 ± 0.01	0.038 ± 0.003	0.9998
	80	1.17 ± 0.03	0.026 ± 0.003	0.9987
	100	1.18 ± 0.07	0.019 ± 0.005	0.9921
GO-3N	20	1.091 ± 0.003	0.34 ± 0.06	1.0000
	30	1.052 ± 0.006	0.52 ± 0.03	0.9999
	40	1.056 ± 0.005	0.27 ± 0.06	0.9999
	60	1.09 ± 0.01	0.07 ± 0.01	0.9997
	80	1.10 ± 0.01	0.043 ± 0.005	0.9996
	100	1.08 ± 0.02	0.03 ± 0.04	0.9988

Adsorption isotherms

The adsorption of Cr(VI) ions on GO modified by amino silanes was also simulated using Langmuir [9,10] and Freundlich [11] isotherm models:

$$q_e = \frac{q_{\max} K_L C_e}{1 + K_L C_e}$$

$$q_e = K_F C_e^{1/n}$$

where q_{max} is the maximum amount of Cr(VI) ions adsorbed per unit weight of modified GO at the high equilibrium ion concentration (mg·g^{-1}), K_L is the constant related to the free energy of adsorption (L·mg^{-1}), and K_F (mg^{1-n}·L^{n}·g^{-1}) and n are Freundlich constants related to the adsorption capacity, respectively. The Langmuir and Freundlich adsorption isotherms are presented in Fig. S4. Isotherm parameters obtained by the fitting of the adsorption equilibrium data to the isotherm models are listed in Table S2. It can be seen that the adsorption isotherms are better fitted by the Langmuir model than by the Freundlich model, suggesting that adsorption of Cr(VI) ions on amino-modified GO nanosheets is monolayer coverage. The maximum adsorption capacity q_{max} values of Cr(VI) on GO-1N, GO-2N and GO-3N are very similar and equal 13.3, 15.1, 14.3 mg·g^{-1}, respectively. Such results are in accordance with SEM/EDS analysis that reveals very similar contents of N in GO-1N, GO-2N and GO-3N, i.e. the same number of active sites on the surface of GO derivatives.

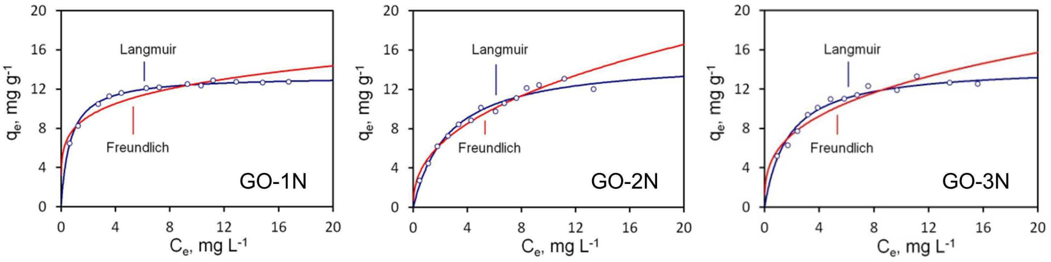

Fig. S4. Langmuir and Freundlich adsorption isotherms; conditions: $m_{adsorbent}$ = 5 mg, pH = 3.5, T = 25 °C, time = 180 min, V = 25 mL.

Table S2. Parameters for Langmuir and Freundlich models of Cr(VI) sorption on GO-1N, GO-2N and GO-3N.

Adsorbent	Langmuir			Freundlich		
	q_{max}	K_L	R^2	K_F	n	R^2
GO-1N	13.3 ± 0.61	1.46 ± 0.079	0.9969	8.0 ± 0.55	5.1 ± 0.28	0.9454
GO-2N	15.1 ± 0.57	0.38 ± 0.035	0.9947	4.8 ± 0.42	2.4 ± 0.20	0.9770
GO-3N	14.3 ± 0.69	0.57 ± 0.040	0.9820	5.9 ± 0.47	3.0 ± 0.27	0.9540

Calibration graph for Cr(VI)

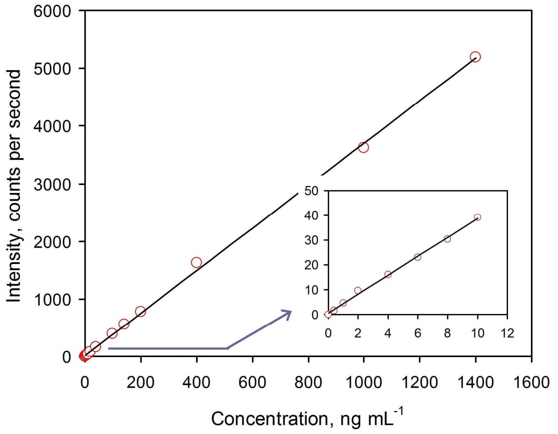

Fig. S5. Calibration graph. Measurement conditions: Cr Kα line, Rh target X-ray tube operated at 20 kV and 450 µA, 120 s counting time, air atmosphere, 200 µm Al primary beam filter.

References

[1] Marcano DC, Kosynkin DV, Berlin JM, Sinitskii A, Sun Z, Slesarev A, Alemany LB, Lu W, Tour JM. (2010) Improved synthesis of graphene oxide. ACS Nano 4:4806–4814

[2] Li Z, Wang R, Young RJ, Deng L, Yang F, Hao L, Jiao W, Liu W (2013) Control of the functionality of graphene oxide for its application in epoxy nanocomposites. Polymer 54:6437–6446

[3] Sitko R, Janik P, Feist B, Talik E, Gagor A (2014) Suspended aminosilanized graphene oxide nanosheets for selective preconcentration of lead ions and ultrasensitive determination by electrothermal atomic absorption spectrometry, ACS Appl. Mater. Interfaces, 6:20144-20153

[4] Liu Z, Duan X, Qian G, Zhou X, Yuan W (2013) Eco-friendly one-pot synthesis of highly dispersible functionalized graphene nanosheets with free amino groups. Nanotechnology 24:045609. https://doi.org/10.1088/0957-4484/24/4/045609

[5] Wu L, Zhang B, Lu H, Liu C-Y (2014) Nanoscale ionic materials based on hydroxyl-functionalized graphene. J Mater Chem A 2:1409–1417

[6] Iqbal MZ, Katsiotis MS, Alhassan SM, Liberatore MW, Abdala AA (2014) Effect of solvent on the uncatalyzed synthesis of amino silane functionalized graphene. RSC Adv 4:6830–6839

[7] Zarrin H, Higgins D, Jun Y, Chen Z, Fowler M (2011) Functionalized Graphene Oxide Nanocomposite Membrane for Low Humidity and High Temperature Proton Exchange Membrane Fuel Cells. J Phys Chem C 115:20774–20781

[8] Ho YS, McKay G (1999) Pseudo-Second Order Model for Sorption Processes. Process Biochem 34:451–465

[9] Langmuir I (1916) The Constitution and Fundamental Properties of Solids and Liquids. J Am Chem Soc 38:2221–2295

[10] Langmuir I (1918) Adsorption of Gases on Plain Surfaces of Glass Mica Platinum. J Am Chem Soc 40:1361–1403

[11] Freundlich HMF (1906) Uber die Adsorption in Lasugen. Z Phys Chem 57:385–470
